# Supplementary material for: The Construction and Exploration of a Comprehensive MicroRNA Centered Regulatory Network in Foxtail Millet (Setaria italica L.)
Source: Front Plant Sci. 2022 May 6;13:848474. doi: 10.3389/fpls.2022.848474 (PMC9121102; doi:10.3389/fpls.2022.848474)
Supplement: Supplementary file 3 [file Data_Sheet_3.zip › Sit-miRN3026a_Seita.3G006300.1_3439_TPlot.pdf]

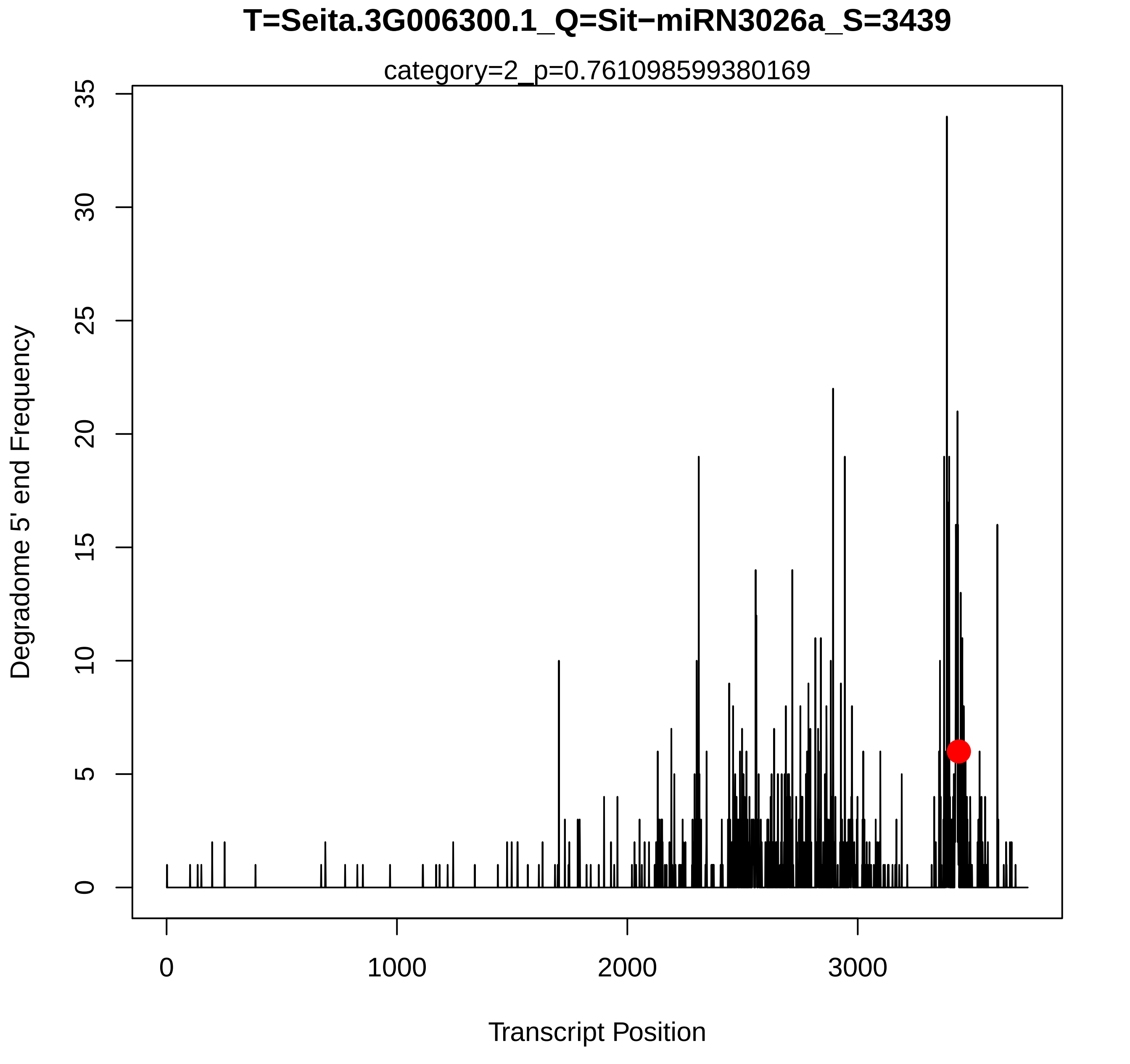
T=Seita.3G006300.1_Q=Sit−miRN3026a_S=3439
category=2_p=0.761098599380169
Degradome 5' end Frequency
0
5
10
15
20
25
30
35
0
1000
2000
3000
Transcript Position
